# Diagnostic performance of a CT-based scoring system for diagnosis of anastomotic leakage after esophagectomy: comparison with subjective CT assessment

**DOI:** 10.1007/s00330-017-4802-3

**Published:** 2017-03-29

**Authors:** Lucas Goense, Pauline M. C. Stassen, Frank J. Wessels, Peter S. N. van Rossum, Jelle P. Ruurda, Maarten S. van Leeuwen, Richard van Hillegersberg

**Affiliations:** 1 Department of Surgery, University Medical Center Utrecht, G.04.228, Heidelberglaan 100, 3584CX Utrecht, The Netherlands; 2 Department of Radiation Oncology, University Medical Center Utrecht, Utrecht, The Netherlands; 3 Department of Radiology, University Medical Center Utrecht, Utrecht, The Netherlands

**Keywords:** Esophagectomy, Anastomotic leakage, Prediction score, Computed tomography, Esophageal cancer

## Abstract

**Objective:**

To develop a CT-based prediction score for anastomotic leakage after esophagectomy and compare it to subjective CT interpretation.

**Methods:**

Consecutive patients who underwent a CT scan for a clinical suspicion of anastomotic leakage after esophagectomy with cervical anastomosis between 2003 and 2014 were analyzed. The CT scans were systematically re-evaluated by two radiologists for the presence of specific CT findings and presence of an anastomotic leak. Also, the original CT interpretations were acquired. These results were compared to patients with and without a clinical confirmed leak.

**Results:**

Out of 122 patients that underwent CT for a clinical suspicion of anastomotic leakage; 54 had a confirmed leak. In multivariable analysis, anastomotic leakage was associated with mediastinal fluid (OR = 3.4), esophagogastric wall discontinuity (OR = 4.9), mediastinal air (OR = 6.6), and a fistula (OR = 7.2). Based on these criteria, a prediction score was developed resulting in an area-under-the-curve (AUC) of 0.86, sensitivity of 80%, and specificity of 84%. The original interpretation and the systematic subjective CT assessment by two radiologists resulted in AUCs of 0.68 and 0.75 with sensitivities of 52% and 69%, and specificities of 84% and 82%, respectively.

**Conclusion:**

This CT-based score may provide improved diagnostic performance for diagnosis of anastomotic leakage after esophagectomy.

***Key Points*:**

• *A CT-based score provides improved diagnostic performance for diagnosis of anastomotic leakage*.

• *Leakage associations include mediastinal fluid, mediastinal air, wall discontinuity, and fistula*.

• *A scoring system yields superior diagnostic accuracy compared to subjective CT assessment*.

• *Radiologists may suggest presence of anastomotic leakage based on a prediction score*.

## Introduction

Esophageal cancer is the sixth leading cause of cancer-related mortality worldwide and the incidence rate is rapidly increasing [1]. Surgical resection of the esophagus with en-bloc lymphadenectomy combined with neoadjuvant chemoradiation or perioperative chemotherapy is the cornerstone of treatment for patients with locally advanced non-metastatic esophageal cancer [2–4]. Despite advances in surgical treatment and improvement in perioperative care, anastomotic leakage remains a frequently encountered complication after esophagectomy with reported frequency rates of up to 30% [2, 5]. Early detection of anastomotic leakage is crucial, since delayed treatment is associated with significant morbidity, prolonged hospital stay, and mortality [6–9].

Several diagnostic modalities are available in case anastomotic leakage is clinically suspected, such as contrast swallow examination, endoscopy, or computed tomography (CT). Contrast swallow examinations are widely performed in order to assess anastomotic integrity. Although contrast swallow examinations are very specific, multiple studies have shown that they are of poor sensitivity, failing to identify significant anastomotic leaks [10–13]. Meanwhile, endoscopy after esophagectomy has proven to be a more accurate method to diagnose anastomotic leakage and provide information on the condition of the gastric tube [14, 15]. However, most physicians are reluctant to utilize endoscopic examination early after esophagectomy as this invasive procedure may damage the anastomosis.

CT scanning is commonly performed for diagnosis of postoperative complications, since it is non-invasive and safe to use in critically ill patients. Previously, several studies have assessed the usefulness of CT scanning for the detection of anastomotic leakage after esophagectomy [13, 16–18]. However, most of these studies assessed the diagnostic value of CT during postoperative routine screening and included only a small number of patients [13, 16, 17]. Also, a wide range of diagnostic accuracies has been reported, suggesting that the association of different radiological findings after esophagectomy with anastomotic leakage is unclear [16–18].

Previous studies have shown that assessment of specific CT findings was useful for the prediction of anastomotic leakage after gastric and colorectal surgery [19, 20].

In summary, objective criteria to detect anastomotic leakage on CT have not been clearly defined. Therefore, the purpose of this study was to determine reliable CT findings that can be used to diagnose anastomotic leakage and develop a CT-based risk prediction score for confirming or ruling out anastomotic leakage in a large cohort of patients with a clinical suspicion of leakage after esophagectomy. Also, the diagnostic performance of this CT-based risk prediction score was compared to that of a systematic subjective evaluation by two expert radiologists and that of the original CT interpretation.

## Materials and methods

### Study population

This retrospective cohort study was approved by an institutional review board and the requirement to obtain informed consent was waived. The study was designed and conducted according to Standards for Reporting of Diagnostic Accuracy [21]. From a prospectively acquired database, all consecutive patients with esophageal or gastro-esophageal junction cancer who underwent an elective esophagectomy, between 2003 and 2014, at our tertiary referral center were identified. Within this database patients who were evaluated with a CT scan for a clinically suspected anastomotic leak after elective esophageal surgery were included. Surgical treatment consisted of a transthoracic or transhiatal esophagectomy with en-bloc lymphadenectomy and gastric tube reconstruction [22]. A cervical esophagogastric anastomosis was performed end-to-side with hand-sewn continuous sutures (3-0 PDS) in monolayer. After surgery two chest tubes were routinely placed, and removed during the following days in case of limited drainage (<200 ml/24 h), and absence of air leak.

### Data collection

Clinical patient characteristics were extracted from the prospectively maintained database (Table 1). In addition, heart rate, temperature, white blood cell count (WBC) and C-reactive protein (CRP) were extracted from the patients’ charts on the day anastomotic leakage was clinically suspected (day of CT scan). Anastomotic leakage was confirmed by either postoperative demonstration of saliva through the cervical wound, or visualization of anastomotic dehiscence or fistula during endoscopy or surgical re-intervention. The follow-up time was truncated to 30 days for all patients. All postoperative complications, including anastomotic leakage, were prospectively registered.

**Table 1:** Clinical and treatment-related characteristics in relation to anastomotic leakage

Characteristic	Anastomotic leakage (*n* = 54)	No anastomotic leakage (*n* = 68)	*p* value
Male gender	41 (75.9)	56 (82.4)	0.382
Age (years)*	65.2 ± 9.0	65.8 ± 8.9	0.708
BMI (kg/m^2^)*	25.7 ± 4.4	26.8 ± 4.3	0.158
ASA score			0.841
I II III IV	12 (22.2) 28 (51.9) 14 (25.9) 0 ( 0.0)	12 (17.6) 40 (58.8) 15 (22.1) 1 ( 1.5)	
COPD	13 (24.1)	10 (14.7)	0.189
Cardiac co-morbidity	15 (27.8)	23 (33.8)	0.474
Diabetes mellitus	9 (16.7)	15 (22.1)	0.457
Current smoker	17 (31.5)	21 (30.9)	0.898
Neoadjuvant therapy			0.973
None Chemotherapy Chemoradiotherapy	16 (30.9) 12 (22.6) 26 (49.1)	21 (29.6) 14 (21.2) 33 (50.0)	
Heart rate*^†^	103 ± 22	96 ± 19	0.085
Temperature*^ǂ^	37.7 ± 0.8	37.7 ± 0.8	0.950
Leukocytes*^§^	16.2 ± 7.1	15.6 ± 7.3	0.385
C-reactive protein*^||^	224 ± 104	194 ± 96	0.110

*Note.* Data are numbers of patients with percentages in parentheses

*Data are mean ± standard deviation

^†^Heart rate in beat per minute

^ǂ^Temperature in Celsius (°*C*)

^§^Number of leukoctyes x 10 ^9^/L

^||^CRP in mg/L

### Image acquisition

Thoraco-abdominal CT images were acquired using commercially available 16- or 64-section CT scanners (Philips Medical Systems, Best, The Netherlands). Images were typically acquired with 64 × 0.625 millimeter section collimation, a tube rotation time of 500 milliseconds, a tube potential of 100 or 120 kV, an effective tube current of 120 mAs, and a pitch of 0.9 or 1.1. An iodinated 90-mL contrast material bolus was administered intravenously at 4 mL/sec in all patients. Oral contrast intake was not routinely used in our center as this was shown to have limited sensitivity for the detection of anastomotic leakage [13].

### Variable selection

CT findings related to anastomotic leakage and esophageal surgery were selected for image analysis by two radiologists and two gastrointestinal surgeons during a consensus meeting. On the basis of their clinical experience, the most frequently encountered CT findings following esophageal surgery and variables described previously in the literature were included for analysis. The selected CT findings included mediastinal fluid collection, mediastinal air, mediastinal abscess (i.e., central zone of necrotic inflammatory material encapsulated by a discernible wall), and mediastinal induration (whenever the mediastinal fat showed non-contiguous, patchy inhomogeneity with water or low-Hounsfield units soft tissue density [<20 Hounsfield units]). When a mediastinal collection was present, the frequency, size of the largest collection, and the anatomical region (i.e., above the manubrium, between the manubrium and carina, between the carina and diaphragm, and below the diaphragm) were recorded. Other CT findings included a visible discontinuity of the esophagogastric wall, a fistula (scored if a fluid- or air-filled tract was visible between the esophagogastric anastomosis and another anatomic cavity [skin, trachea, pleural cavity or mediastinum]), pleural effusion, empyema (atypical pleural effusion, loculation in the pleural space, and thickening of the pleural membranes) and presence of a lung consolidation (i.e., atelectasis, pneumonia or non-specific).

### Image evaluation

All CT scans were retrospectively reviewed together by two radiologists in consensus (MSL and FJW with more than 25 and 6 years experience in gastrointestinal imaging, respectively). Images were reviewed on a picture archiving and communication system (Sectra AB, version 17.3, Linköping, Sweden). The reviewers knew that all patients had been subjected to an esophagectomy, but were blinded for the patients’ detailed clinical information. The presence or absence of the various selected CT variables were systematically assessed and recorded. After the systematic assessment of the CT findings, the reviewers also indicated if they suspected the patient to have an anastomotic leak (i.e., no anastomotic leak, a probable leak, or a definite anastomotic leak), further referred to as the systematic subjective assessment.

Also, all the original CT interpretations rendered as part of the clinical care were reviewed. Each CT report was originally interpreted by a board certified radiologist. The original interpretations were classified as ‘no leak, ‘probable leak’, or ‘definite leak’.

### Statistical analysis and development of a practical scoring system

The association of clinical patient characteristics with anastomotic leakage was studied univariably. Categorical parameters were compared using the chi-square test or Fisher’s exact test in case of small cell counts. Student’s T-test and the Mann-Whitney-U test were used to compare groups with and without anastomotic leakage for parametric and non-parametric continuous parameters, respectively. In order to analyze whether the different CT findings were associated with anastomotic leakage, univariable logistic regression models were used providing odds ratios (ORs) with 95% confidence intervals (CIs). Subgroup analyses were performed to assess whether location in the mediastinum and days after surgery influenced the associations of the different CT findings with anastomotic leakage.

Subsequently, parameters with a *p*-value below 0.05 in univariable logistic regression analysis were entered into a multivariable logistic regression model with backward stepwise selection to evaluate whether these factors were independently associated with the occurrence of anastomotic leakage. A practical scoring system was developed using the *beta*-regression coefficients of the retained predictive factors.

To compare diagnostic performances of the different CT assessments, receiver operating characteristics (ROC) curve analysis was performed and the area-under-the-curves (AUC) were computed. Ideal cut-off values were calculated by giving equal weight to sensitivity and specificity. In addition, the potential superiority of the prediction score in comparison with the systematic subjective and original assessment was evaluated using the net reclassification index (NRI). The NRI reflects the reclassification ability of the model and is the sum of improvement in correctly predicting patients with and without leakage [23]. Statistical analysis was performed using SPSS version 23.0 (IBM Corp., Armonk, NY). A *p*-value of <0.05 was considered statistically significant.

## Results

### Demographics

**Fig. 1:**
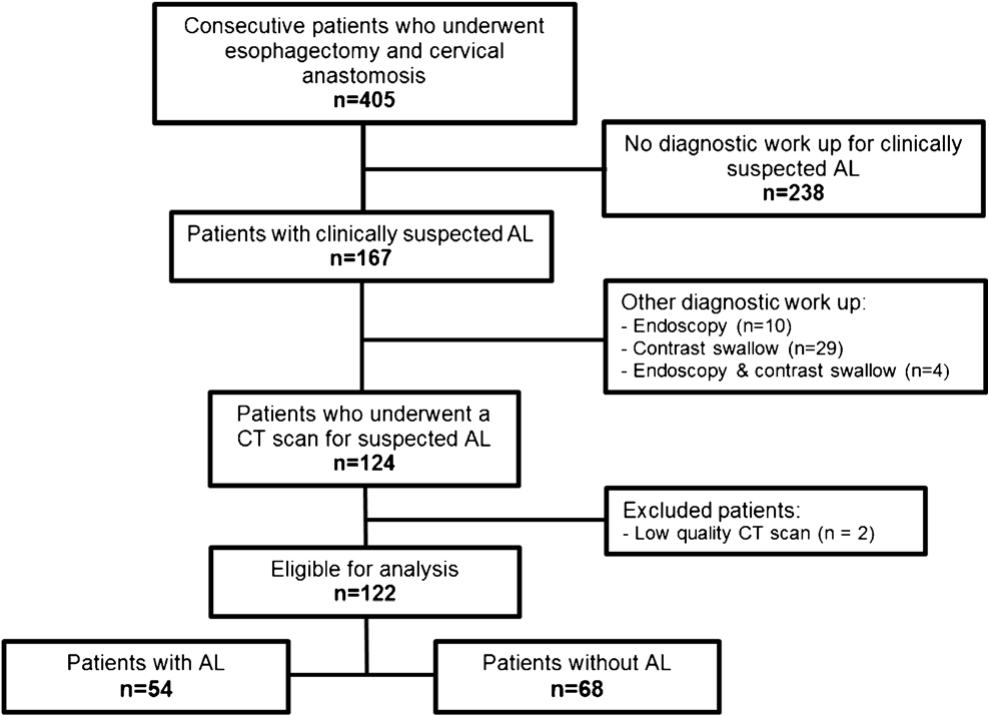
Flowchart demonstrates the selection process of patients with a suspicion of anastomotic leakage (AL)

In the study period, a total of 405 patients underwent esophagectomy with gastric tube reconstruction. Of these patients, 283 were excluded because there was no clinically suspected leak (*n* = 238), no CT scan was performed in case of a suspected anastomotic leak (*n* = 43) or the CT scan was of insufficient quality (*n* = 2). Consequently, 122 patients were deemed eligible for inclusion in our study, of whom 54 (44.3%) had a confirmed anastomotic leak (Fig. 1).

Clinical and treatment-related patient characteristics and their univariable association with anastomotic leakage are summarized in Table 1. None of the studied patient and treatment-related factors were significantly associated with the occurrence of anastomotic leakage. The median time interval between esophagectomy and CT acquisition was 6 days (range: 1-32). Anastomotic leakage occurred after a median time of 8 days (range: 1-22) following esophagectomy. Anastomotic leakage was confirmed by, endoscopy (*n* = 10), surgical re-intervention (*n* = 31) or demonstration of saliva during opening of the cervical wound (*n* = 13). Treatment of anastomotic leakage consisted of ceasing oral intake in combination with opening of the cervical wound (*n* = 20), placing a stent (*n* = 3) or surgical re-intervention (*n* = 31).

### Predictors of anastomotic leakage

The results of univariable logistic regression analyses for each specific CT finding in relation to anastomotic leakage are presented in Table 2. In univariable analyses studying specific CT findings, the presence of a mediastinal fluid collection (OR 3.1, 95% CI: 1.4–7.1, *p* = 0.006) and mediastinal air (OR 11.1, 95% CI: 3.6–34.2, *p <* 0.001) were significantly associated with anastomotic leakage.

**Table 2:** Univariable logistic regression analysis of specific postoperative CT findings in relation to anastomotic leakage after esophagectomy

Characteristic	Anastomotic leakage (*n* = 54)	No anastomotic leakage (*n* = 68)	OR (95% CI)	*p* value
Mediastinal:				
Induration	7 (13.0)	6 ( 8.8)	1.5 (0.49-4.88)	0.464
Fluid collection	23 (42.6)	13 (19.1)	3.1 (1.40-7.06)	0.006*
Abscess	7 (13.0)	4 (5.9)	2.4 (0.66-8.61)	0.185
Air	50 (92.6)	36 (52.9)	11.1 (3.61-34.20)	<0.001*
Wall discontinuity	27 (50.0)	5 (7.4)	12.6 (4.39-36.20)	<0.001*
Fistula	15 (27.8)	2 (2.9)	12.7 (2.76-58.47)	<0.001*
Pleural effusion	46 (85.2)	58 (85.3)	1.0 (0.36-2.71)	0.987
Empyema	11 (20.4)	1 (1.5)	17.1 (2.1-137.6)	0.007*
Atelectasis	50 (92.6)	59 (86.8)	1.9 (0.55-6.57)	0.306
Pulmonary infiltrate	11 (20.4)	19 (27.9)	0.7 (0.28-1.54)	0.336

Note – Data presented as counts with percentages in the parentheses

*Significant difference between patients with versus without anastomotic leakage (*p* < 0.05)

OR: odds ratio. CI: confidence interval

In subgroup analyses the associations of mediastinal fluid with anastomotic leakage was independent of its size, anatomic location within the mediastinum, and the number of days they occurred after surgery. In subgroup analyses of patients with presence of mediastinal air on their postoperative CT scan (70%, 86/122), the number of days after surgery that air was observed was significantly associated with anastomotic leakage (OR for each additional postoperative day: 1.162, 95% CI: 1.022–1.327, *p* = .022). To this regard, the prevalence of a confirmed leak in patients with observed free air before or after the seventh postoperative day was 50% and 73%, respectively. Of the patients with free air in the mediastinum, air was observed above the manubrium in 22 patients (12/22, 55% leakage), between the manubrium and carina in 4 patients (3/4, 75% leakage), between carina and diaphragm in eight patients (4/8, 50% leakage), and in a combination of these anatomic locations in 52 patients (31/52, 60% leakage). The association of mediastinal air with anastomotic leakage was independent of its location (*p* = 0.838). Also the size of mediastinal air on CT was not associated with anastomotic leakage.

In addition, the presence of wall discontinuity (OR 12.6, 95% CI: 4.39–36.20, *p* < 0.001), fistula (OR 12.7, 95% CI: 2.8–58.5, *p* < 0.001) and empyema (OR 17.1, 95% CI: 2.1–137.6, *p* = 0.007) were significantly associated with anastomotic leakage. No significant difference in incidence of other CT findings among patients with or without anastomotic leakage was found.

In multivariable logistic regression analysis, a mediastinal fluid collection (OR 3.4, 95% CI: 1.3–9.4, *p* = 0.016), mediastinal air (OR 6.6, 95% CI: 1.9–23.2, *p* = 0.003), wall discontinuity (OR 4.9, 95% CI: 1.5–15.9, *p* = 0.008), and presence of a fistula (OR 7.2, 95% CI: 1.2–43.8, *p* = 0.032) remained independently and significantly associated with anastomotic leakage (Table 3, Fig. 3). The association between empyema and anastomotic leakage was no longer significant after multivariable adjustment (*p* = 0.093).

**Table 3:** Multivariable logistic regression analysis of CT findings significantly associated with anastomotic leakage in univariable analysis

Characteristic	β regression coefficient	OR (95% CI)	*p* value	Points^†^
Fluid collection	1.233	3.43 (1.26-9.34)	0.016*	1
Air cavity	1.882	6.57 (1.86-23.21)	0.003*	1
Wall discontinuity	1.591	4.91 (1.52-15.88)	0.008*	1
Fistula	1.973	7.19 (1.18-43.84)	0.032*	1
Empyema	1.987	7.29 (0.72-74.30)	0.093	NA

* Significant difference between patients with versus without anastomotic leakage (*p* < 0.05)

OR: odds ratio, CI: confidence interval

^†^Assignment of points to CT findings was based the corresponding β regression coefficient. Scaling was performed with respect to the discriminatory power of the scores as determined by ROC analysis

### Systematic subjective CT assessment

**Fig. 2:**
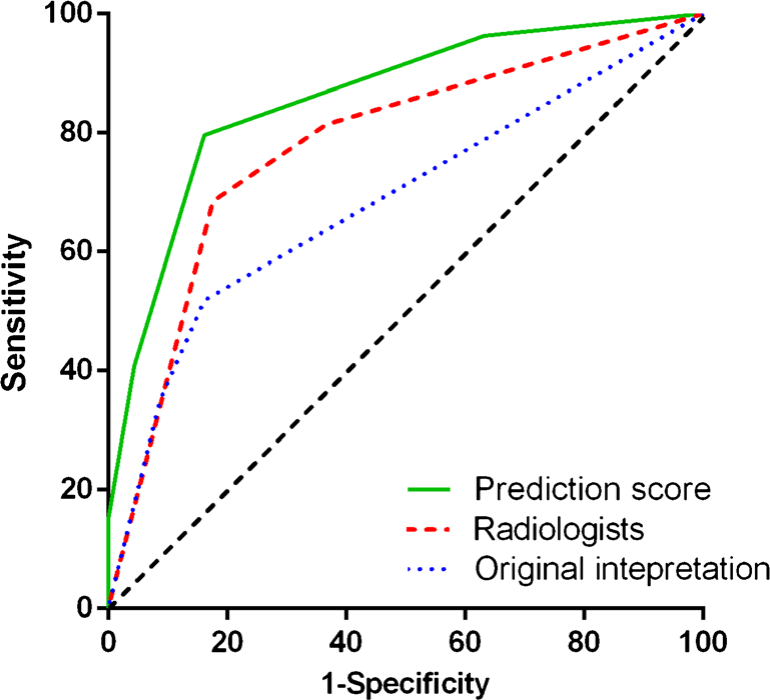
ROC curve analysis of the ‘anastomotic leakage prediction score’ (green line), the systematic subjective CT assessment by expert radiologists (red dotted line) and the original interpretation (blue dotted line) indicating their ability to discriminate between patients with and without anastomotic leakage

During systematic subjective CT scan assessment by the radiologists, a leak was suggested in 49 patients of which 37 (75.5%) had a confirmed leak, whereas absence of a leak was scored in 53 patients of which ten (18.9%) had a confirmed leak. Of the remaining patients with a probable leak (*n* = 20), 7 (35%) had a confirmed leak. The radiologists evaluation, referred to as ‘systematic subjective assessment’, yielded an AUC of 0.75 (95% CI: 0.66–0.84) in ROC analysis (Fig. 2, Table 4). Sensitivity and specificity of the systematic subjective assessment by the radiologists (no + probable leakage versus presence of leakage) were 68.5% (37 of 54; 95% CI: 54.3-80.1) and 82.4% (56 of 68; 95% CI: 70.8-90.1), respectively (Table 4).

**Table 4:** Receiver operating characteristics analysis and net reclassification index (NRI) estimates for anastomotic leakage according to the original interpretation, subjective CT assessment and the anastomotic leakage prediction model

Model	AUC (95% CI)	Ideal cut-off	SE (%)	SP (%)	PPV (%)	NPV (%)	NRI (%)
Original CT interpretation	0.68 (0.59-0.78)	No AL vs. probable or definite AL	51.9	83.8	71.8	68.7	reference
Systematic assessment	0.75 (0.66-0.84)	No or probable AL vs. definite AL	68.5	82.4	75.5	76.7	15.2
ALP-score model	0.86 (0.79-0.92)	Score ≥2 vs. score <2	79.6	83.8	81.1	83.8	27.7

ALP-score: anastomotic leakage prediction score. AL: anastomotic leakage. AUC: area under the curve. SE: sensitivity. SP: specificity. PPV: positive predictive value. NPV: negative predictive value. NRI: Percentage of net reclassification index

### Original clinical CT interpretation

The original clinical CT interpretation yielded an AUC of 0.68 (95% CI: 0.59-0.78) in ROC analysis (Fig. 2, Table 4). Sensitivity and specificity of the original assessment by the radiologists (absence of leakage versus probable + presence of leakage) were 51.9% (28 of 54; 95% CI: 38.0-65.5) and 83.8% (57 of 68; 95% CI: 72.5-91.3), respectively (Table 4).

### Risk scoring system

An anastomotic leakage prediction score (ALP score) was constructed based on the four CT findings that remained significantly associated with anastomotic leakage in multivariable analysis. Based on the absolute *beta-*regression coefficient, presence of each variable was converted into a corresponding amount of points rounded to its nearest integer. Next scaling was performed with respect to the discriminatory power of the scores as determined by ROC analysis. To this regard it proved feasible to assign one point for the presence of each predictive factor – in order to keep the score simple – without compromising its discriminative ability (Table 3). Therefore, the cumulative amount of points of the ALP score ranges from 0 to 4. The diagnostic performance of the possible scores for identifying an anastomotic leak are presented in Table 5.

**Table 5:** Risk scores and their coordinates on the ROC curve

Risk score	n	Observed leakage risk	Sensitivity* (%)	Specificity* (%)
Anastomotic leakage prediction score				
ALP score 0	27	7.4% (2/27)	100	0
ALP score 1	41	22.0% (9/41)	96.3	36.8
ALP score 2	29	72.4% (21/29)	79.6	83.8
ALP score 3	17	82.4% (14/17)	40.7	95.6
ALP score 4	8	100% (8/8)	14.8	100
Systematic subjective assessment				
No leakage	53	18.9% (10/53)	100	0
Probable leakage	20	35.0% (7/20)	81.5	63.2
Presence of leakage	49	75.5% (37/49)	68.5	82.4
Original CT interpretation				
No leakage	83	31.3% (26/83)	100	0
Probable leakage	14	64.3% (9/14)	51.9	83.8
Presence of leakage	25	76.0% (19/25)	35.2	91.2

ROC: receiver operating characteristics

* Sensitivity and specificity defined by their coordinates on the ROC curve

Using ROC analysis a total of two points was statistically determined as optimal cut-off, in which patients with scores ≥2 points were considered at high risk of anastomotic leakage. The cut-off value of ≥2 points yielded a sensitivity of 80% (43 of 54; 95% CI: 66.1–88.9) and specificity of 84% (57 of 68; 95% CI: 72.5–91.3). The final ALP score model had an AUC of 0.86 (95% CI: 0.79-0.93) (Fig. 2, Table 4).

**Fig. 3:**
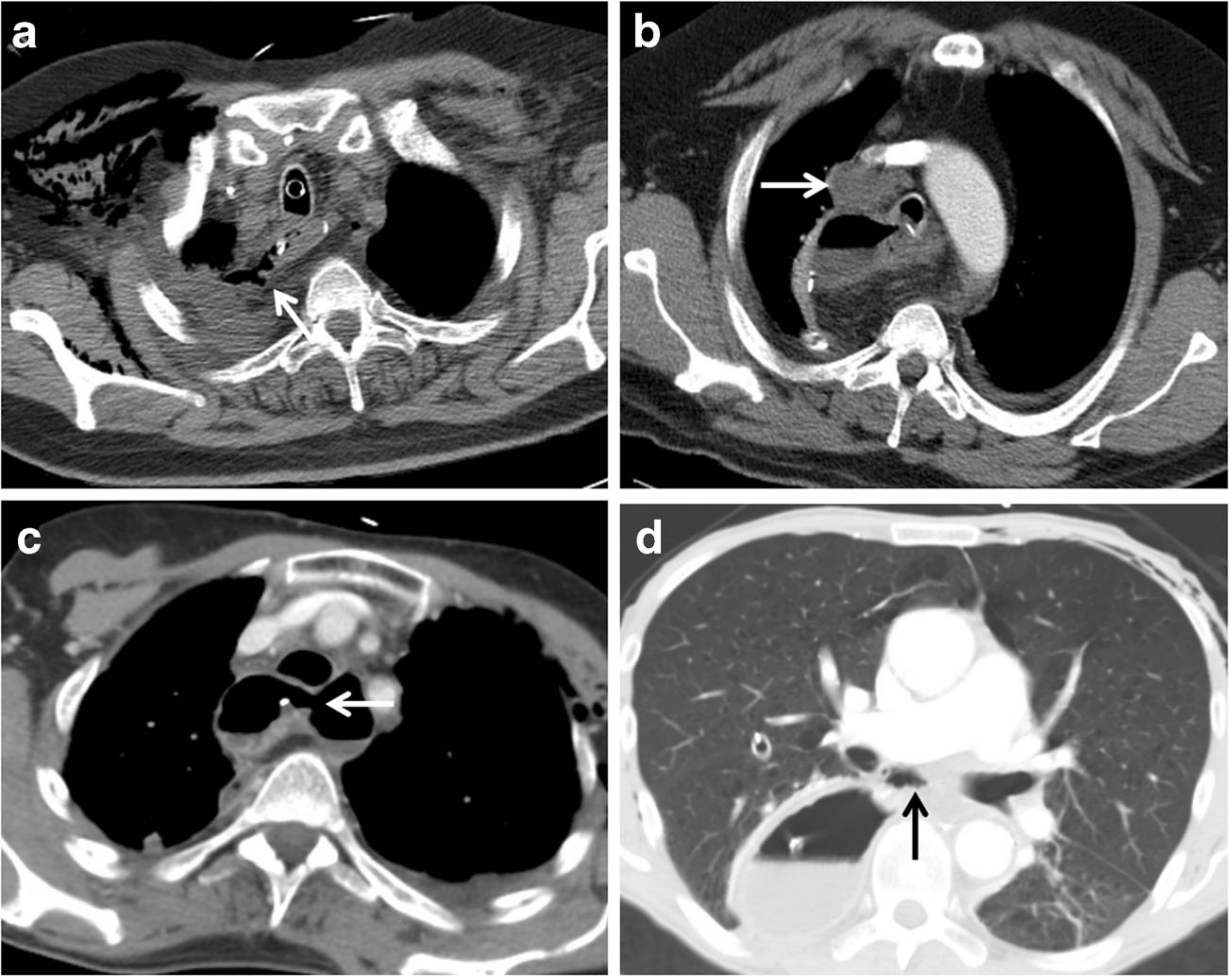
Examples of CT findings associated with the presence of anastomotic leakage after esophagectomy. **A**: Image shows a fistula between the gastric tube and right pleural cavity (*arrow*). **B**: Image shows a fluid collection (*arrow*) in the mediastinum. **C**: Image shows a visible discontinuity of the esophagogastric wall (*arrow*). **D**: Image shows a mediastinal air cavity (*arrow*) after esophagectomy

The ALP scoring system improved the AUC (0.86 versus 0.75 and 0.68) with an NRI of 12.5% (*p* = 0.008) and 27.7% (*p* < 0.001) for the detection of anastomotic leakage compared to the systematic subjective CT assessment and original CT interpretation, respectively (Table 4). These findings indicate that with the ALP score 11.1% and 27.7% of the patients with definite anastomotic leakage, and 1.4% and 0% of patients without leakage were better classified compared to the systematic subjective CT assessment and original CT interpretation, respectively (Fig. 3).

## Discussion

In this retrospective cohort study, CT findings of patients with a clinically suspected anastomotic leak after esophagectomy were systematically analyzed and predictors of anastomotic leakage were identified. Presence of mediastinal fluid, mediastinal air, esophagogastric wall discontinuity and fistula were independently associated with anastomotic leakage, irrespective of clinical and treatment-related patient characteristics. Based on these CT findings, a prediction score for anastomotic leakage was developed. The 4-point ALP score demonstrated good diagnostic performance. This study demonstrates superior diagnostic accuracy of a CT-based scoring system in comparison with the systematic subjective assessment (NRI: 12.5%), and original CT interpretation (NRI: 27.7%) of leakage on a post-esophagectomy CT scan. The easy to use point-based ALP score may provide radiologists and surgeons a tool to objectively assess the risk of anastomotic leakage after esophageal surgery in patients with a suspicion of such complication.

CT scanning is increasingly being performed for the detection of anastomotic leakage after esophagectomy, since it is non-invasive, safe in critically ill patients, and aids in the detection of other associated findings (e.g., pulmonary complications) [16, 17]. However, the interpretation of a CT scan after prior esophageal resection remains difficult due to the anatomic changes and residual air and fluid shortly after surgery. Previous studies have assessed the diagnostic value of CT scanning for the detection of anastomotic leakage after esophagectomy [13, 16–18]. In most of these studies, one or two reviewers determine their own definition as to what an anastomotic leak on a CT scan consists of, without assessing specific CT findings [16, 17]. In the literature this results in a large difference in reported diagnostic values [16, 17]. This observation is confirmed by the current study in which a difference of 17% in sensitivity was found between the original CT interpretation and the systematic subjective CT assessment. These findings are suggestive for a lack of consensus on radiographic findings associated with anastomotic leakage in patients after esophagectomy.

Until now, two studies have made a similar attempt to identify specific CT findings for the detection of anastomotic leakage after esophagectomy [13, 18]. One study that included 97 patients assessed several specific CT findings during postoperative routine screening. In that study presence of mediastinal air and contrast leakage on postoperative day 3 and 7 were associated with anastomotic leakage [13]. Another study that included 54 patients found mediastinal air and mediastinal fluid to be associated with anastomotic leakage [18]. These observations partially correspond with the results of our study that found an association of anastomotic leakage with mediastinal air and fluid collections. However, in these two previously mentioned studies only few events of anastomotic leakage occurred (*n* = 11 and *n* = 6, respectively), which results in an uncertainty of estimates [13, 18]. Interestingly, these studies show that using solitary CT findings as diagnostic marker, without combining them in a model, results in either low sensitivity or low specificity. Contrast leakage after esophagectomy, for example, is known to be a specific finding but the absence of extravasation of contrast is associated with high false-negative rates and consequently a low sensitivity [13]. On the contrary, presence of mediastinal air near the gastric tube is very sensitive, but since this is a common finding after esophageal surgery it is not very specific [13, 18]. Our data suggest that combining specific CT findings in a risk score could be used to overcome these limitations and improve diagnostic accuracy of CT scanning after esophagectomy.

The developed ALP score has a good predictive value and includes well-recognized CT findings. The data indicate that, in the presence of two or more of four CT findings, the decision whether to start treatment could be made quite reliably, without true additional value of other diagnostic tests. This could lead to a reduction in treatment delay that is associated with additional tests. Although the cut-off point of ≥2 yielded the highest overall discriminatory value, each additional point was associated with an increased risk, and clinical reasoning (particularly with scores of 1 and 3) remains important for treatment decision-making. To this regard, endoscopy after esophagectomy may be useful in cases where the results of the CT-scan are uncertain. Endoscopy has proven to be an accurate method to diagnose anastomotic leakage [14, 15].

In the current study in appeared counterintuitive that empyema fell out of the multivariable model, as it was highly predictive in univariable analysis. However, in multivariable prediction modeling the outcome (anastomotic leakage) is predicted based on values of a set of predictor variables (CT parameters). This method allows us to assess the impact of multiple predictor variables in the same model. In the current series at least two or more predictor variables that were highly suggestive for anastomotic leakage (i.e., fluid collection, air cavity, wall discontinuity, and fistula) were present in all 12 patients with empyema. Therefore, the added value of empyema - over the other predictor variables - for the prediction of anastomotic leakage was redundant in the current model, and therefore lost its significance. However, the fact remains that in clinical practice the presence of empyema on a postoperative CT scan is highly suggestive for the presence of an anastomotic leak.

Various limitations apply to this study. First, this study is limited by its retrospective nature. Second, the specific CT findings may be subject to interobserver variability. Standardization of mediastinal CT findings may overcome this problem. Finally, external validation of the prediction score is warranted since there is a risk of model overfitting due to multiple testing and differences with other patient populations (e.g., prevalence of leakage, use of other surgical techniques).

In conclusion, this study demonstrates that the presence of mediastinal fluid, mediastinal air, esophagogastric wall discontinuity and a fistula on a postoperative CT scan are independently and significantly associated with anastomotic leakage after esophagectomy in patients with a clinical suspicion of anastomotic leakage. Based on these items a CT-based anastomotic leakage prediction score was developed with superior discriminatory ability compared to systematic subjective CT assessment and original CT interpretation for the detection of anastomotic leakage after esophagectomy.

## Abbreviations

ALP-score: anastomotic leakage prediction score
AUC: area-under-the-curve
CI: confidence interval
CT: computed tomography
CRP: C-reactive protein
NRI: net reclassification index
OR: odds ratio
WBC: White blood cell count
